# A Cohort Study on Factors Affecting Sleep Quality in Women Undergoing Intrauterine Sperm Insemination (IUI) Treatment

**DOI:** 10.1155/ogi/4749954

**Published:** 2025-07-28

**Authors:** Zahra Vafaeian, Maryam Hassanzadeh Bashtian, Tooba Farazmand, Alireza Afshari-Safavi, Seyed Kaveh Hojjat, Sepideh Hamdamiyan

**Affiliations:** ^1^School of Medicine, North Khorasan University of Medical Sciences, Bojnurd, Iran; ^2^Addiction Research Center and Behavioral Sciences, North Khorasan University of Medical Sciences, Bojnurd, Iran; ^3^Department of Midwifery, School of Nursing and Midwifery, North Khorasan University of Medical Sciences, Bojnurd, Iran; ^4^Department of Gynecology, School of Medicine, North Khorasan University of Medical Sciences, Bojnurd, Iran; ^5^Department of Statistics and Epidemiology, School of Health, North Khorasan University of Medical Sciences, Bojnurd, Iran; ^6^Division of Sleep Medicine, Psychiatry and Behavioral Sciences Research Center, Mashhad University of Medical Sciences, Mashhad, Iran; ^7^Bent-Al-Hoda Hospital, North Khorasan University of Medical Sciences, Bojnurd, Iran

**Keywords:** anxiety, depression, infertility, IUI, sleep disorder

## Abstract

**Introduction:** The stress associated with infertility can impact an individual's sleep status by affecting the hypothalamus and pituitary axis, potentially leading to sleep disorders. On the other hand, sleep disorders can further contribute to the development of depression and anxiety. This study aims to investigate the factors that influence sleep quality in women undergoing intrauterine sperm insemination (IUI) treatment.

**Methods:** This research involved a prospective cohort study conducted on 131 infertile women aged 18–45 years who sought services at the infertility clinic of Bent Al-Hoda Hospital in Bojnurd City and a private clinic between 2020 and 2023. Data were gathered using a demographic questionnaire, the Beck Anxiety Inventory (BAI), the Beck Depression Inventory (BDI), and the Pittsburgh Sleep Quality Index (PSQI). Participants completed these questionnaires at three different time points: 0, 14, and 30 days in relation to the time of IUI. Data analysis was carried out using repeated-measures analysis of variance and generalized linear models.

**Results:** The average age of the participating women was 29.85 years, with a standard deviation of 5.75. The overall prevalence of sleep quality disorder was 30.5%. Most patients reported mild to moderate disturbances in the delay of falling asleep. While no significant difference was observed in the comparison of average sleep disturbance scores at three different times, an increasing trend in anxiety and depression was noted in the second stage, followed by a decreasing trend in the third stage. In the presence of other variables, anxiety and depression demonstrated a significant relationship with sleep disorder (*p* < 0.001).

**Conclusions:** Approximately one-third of infertile women were found to be suffering from a sleep quality disorder. The study underscores the significant impact of depression and anxiety on sleep quality disorders among infertile women. As a recommendation, it is advised to address the psychological well-being of infertile patients within infertility treatment clinics.

## 1. Introduction

Infertility is clinically defined as the inability to conceive after 1 year of frequent unprotected intercourse or after 6 months if the woman is over 35 years old. It is categorized into primary infertility and secondary infertility [1, 2]. Beyond its medical implications, infertility is a social condition, representing a chronic and low-pressure stressor linked to enduring negative social and psychological consequences. The attainment of pregnancy/birth through assisted reproductive technology (ART) treatment is linked to heightened psychological well-being, while treatment failure is associated with increased levels of anxiety and depression during and after the treatment period [3].

In the realm of infertility treatment, intrauterine sperm insemination (IUI) stands out as a common method [4]. This approach is particularly beneficial for patients with mild male factor infertility, anovulation, endometriosis, and unexplained infertility [5]. Studies indicate that approximately 65% of individuals undergoing IUI reported significant anxiety, with about 17% experiencing depression [4].

In a related study addressing the psychological challenges faced by infertile women, the findings revealed that the average depression score among these women was 32.01 ± 12.49, contrasting with 21.85 ± 10.98 observed in fertile women. Additionally, the average anxiety score for infertile women was reported as 51.36 ± 12, compared to 36.20 ± 9.63 for their fertile counterparts [6].

Sleep plays a crucial role in overall health and well-being, influencing various aspects of physical and mental health. Sleep-related issues are associated with an increased risk of conditions such as high blood pressure, diabetes, obesity, depression, heart attack, and stroke [7]. Sleep disorders encompass both short sleep duration and poor sleep quality, with short sleep duration defined as getting 6 h of sleep or less [8]. The prevalence of sleep disorders tends to increase with age, and women are more commonly affected than men. Furthermore, sleep disorders are often linked to fluctuations in fertility hormones [9]. Specifically, in the realm of female reproductive health, sleep disorders have been associated with postpartum depression, the transition to menopause, and premenstrual symptoms [10].

Studies conducted by Lin et al. highlight that infertile women undergoing assisted reproductive treatments may experience significant hormonal changes, leading to physical complaints, psychological distress, and sleep disorders. Lin's research, which utilized the Pittsburgh Sleep Quality Index (PSQI), found that sleep quality was poor in 35% of women undergoing infertility treatment with intrauterine insemination [11]. The results of a systematic review by Li et al. (2024) on sleep patterns, sleep disorders, sleep-disordered breathing, and their association with infertility or female fertility revealed that poor sleep quality, extremely short or long sleep durations, and certain sleep chronotypes are linked to poorer outcomes in fertility treatments. These outcomes include a reduced number of retrieved oocytes, lower embryo quality, and decreased fertilization rates. Similarly, a systematic review and meta-analysis by Habibi et al. found that sleep quality is significantly associated with in vitro fertilization (IVF) outcomes, such as pregnancy rates [12, 13]. However, there is a scarcity of studies investigating factors influencing sleep disorders in infertile women, and no similar study has been conducted to date. Given the stressful nature of infertility and its treatment stages, which can adversely impact treatment success, timely intervention and referral to mental health specialists can potentially assist infertile women. Therefore, the objective of this study was to identify the factors influencing sleep quality in infertile women undergoing IUI treatment.

## 2. Materials and Methods

The study conducted was a prospective cohort study involving 131 infertile women aged 18–45 years. The participants were recruited from the infertility clinic of Bent Al-Hoda Hospital, the primary infertility clinic in North Khorasan province, and a private infertility clinic in Bojnurd between 2020 and 2023.

The sample size determination utilized a mean estimation formula, with the PSQI score estimation derived from the Shahraki study [14]. The anticipated average sleep quality score was 4.7 with a standard deviation of 2.6. The error rate was set at 0.05, and the study's precision (d) was established at 0.52. Using the formula, the initial sample size was calculated to be 100 individuals. To enhance the study's accuracy and account for potential patient dropouts, the final sample size was set at 131 people.

(1) $$\begin{array}{c} n=\frac{{\left(z1-\left(\alpha /2\right)\right)}^{2}{\left(\text{SD}\right)}^{2}}{d^{2}}. \end{array}$$

The inclusion criteria for the study encompassed infertile women between the ages of 18 and 45 who were candidates for IUI treatment. These individuals were required to have an absence of known mental illness such as severe anxiety, depression or psychosis in the last 6 months, and not taking antianxiety and antidepressant drugs and sleeping pills in the last 6 months. Literacy and Iranian nationality were also considered as inclusion criteria.

On the other hand, exclusion criteria involved participants who expressed unwillingness to continue participating in the research, experienced a severe physical illness during the research stages (e.g., severe COVID-19 or an accident), or encountered any circumstance affecting their mental state, and/or an emotional or mental shock during the study duration (e.g., the death of a relative).

Several questionnaires were utilized to gather information, including the demographic questionnaire, the Beck Anxiety and Depression Inventories, and the PSQI. Participants completed these questionnaires at three distinct time points: 0, 14, and 30 days in relation to the time of IUI. The data collected were subjected to analysis using SPSS Version 20, with a significance level set at 0.05.

To capture personal and social characteristics, a demographic information questionnaire was employed. This questionnaire covered details such as age, education, occupation, duration of marriage, duration, cause, and type of infertility, and history of gynecological and obstetric diseases. Additionally, information about the use of traditional and herbal medicines was included in the demographic questionnaire.

The PSQI is a comprehensive tool that evaluates various aspects of an individual's sleep. It encompasses seven dimensions: subjective quality of sleep, delay in falling asleep, sleep duration, sleep efficiency, sleep disorders, use of sleeping pills, and daily functional disorders. Each of these dimensions is scored between 0 and 3, indicating the absence of sleep problems, moderate sleep problems, serious sleep problems, and very serious sleep problems, respectively. The overall sleep quality score is derived from the sum of the scores of all subscales, resulting in a number between 0 and 21. A score exceeding 5 is indicative of the presence of a sleep disorder. In the study by Buysse et al., a total score on the PSQI above 5 showed a diagnostic sensitivity of 89.6% and a specificity of 86.5%. The kappa coefficient for distinguishing between good and poor sleepers was 0.75, which was statistically significant (*p* < 0.001) [15]. In a study conducted by Moghaddam et al., the sensitivity and specificity for distinguishing insomnia patients from controls were 94% and 72%, respectively, at a PSQI cutoff value of 5, and 85% and 84%, respectively, at a cutoff value of 6 [16]. Thus, the PSQI demonstrates acceptable validity and reliability.

In this study, the 21-item version of the Beck Depression Inventory (BDI) was employed. This version, an updated and revised model of the original Beck questionnaire, encompasses cognitive, motivational, emotional, physiological, and other factors contributing to the assessment of depression. Numerous studies have scrutinized and confirmed the questionnaire's validity, reliability, factor analysis, and cutoff points. Each question offers four options, graded from 0 to 3, with a higher score indicating greater severity of depression. Respondents answer 21 questions regarding their feelings in the last two weeks until the present. The maximum score achievable on this questionnaire is 63. As a general guideline, a score of 19–14 indicates mild depression, 20–28 suggests moderate depression, and 29–63 signifies severe depression [17]. In a study conducted by Ghassemzadeh et al. in Iran (2005), the BDI demonstrated a high internal consistency with a Cronbach's alpha coefficient of *α* = 0.87 [18].

Beck's Anxiety Inventory comprises 21 questions with four options, resulting in a score range of 0–63. Scores categorize participants into one of four groups: no anxiety (score less than 9), mild anxiety (score 10–18), moderate anxiety (score 19–29), and severe anxiety (score 30–63). Responses are scored as follows: zero for the natural option, one for mild, two for moderate, and three for severe [19]. The scientific validity of the translated version in Iran was established by Hashemiyeh Chehr through face validity and content validity. Cronbach's alpha coefficient for internal correlation was found to be *α* = 0.85, indicating strong internal consistency in measuring anxiety [20].

The study spanned 2 years and 9 months, during which 290 patients were initially included. Ultimately, 131 patients completed all three stages of the questionnaire.

### 2.1. Ethics Approval Statement

This research was approved by project code 980216 and ethics code number IR.NKUMS.REC.1399.051 in North Khorasan University of Medical Sciences.

### 2.2. Patient Consent Statement

The necessary information to access the samples was obtained from the centers, and after contacting the eligible individuals to enter the research, explaining the objectives of the project, and obtaining informed consent, necessary arrangements were made for questionnaire completion.

The statistical analysis methods employed in the study included both descriptive and inferential techniques. Descriptive statistics were utilized to present data characteristics, including central indices such as mean and median, and dispersion indices like standard deviation and range. To compare the averages of sleep disorder, depression, and anxiety across the three stages, repeated-measures analysis of variance was conducted. Additionally, the relationship between demographic factors, anxiety, and depression with sleep disorder was explored through the use of the generalized linear model (GLM). The significance level for the data analysis was set at 0.05. All data analysis procedures were performed using SPSS Version 20 software.

## 3. Results

The average age of the women participating in the study was 29.85 years, with a standard deviation of 5.75 years. The majority of the women and their husbands had attained a university-level education. Notably, a significant proportion of the women (83.1%) were unemployed, and a majority of the husbands were engaged in freelance jobs. Ovulation disorders emerged as the most prevalent cause of infertility, accounting for 37.3% of cases, and the majority of patients experienced primary infertility (72.9%). The average duration of infertility was reported to be 4.85 years, with a standard deviation of 3.39 (Table 1).

In the review of the first stage of the sleep disorder questionnaire, the overall prevalence of sleep quality disorder in the patients studied in this research was 30.5%. A detailed examination of the seven domains of sleep quality revealed that the majority of patients did not exhibit disorders in the areas of sleep duration, sleep efficiency, and daily dysfunction. Additionally, most patients did not use sleep-inducing drugs. In terms of subjective quality of sleep and sleep disorders, the majority of patients experienced mild disorders. Specifically, patients reported mild to moderate disturbances in the domain of delay in falling asleep. Only one patient had a severe disorder in the area of taking sleeping pills and sleep disorders.

In the initial evaluation using the depression questionnaire, the prevalence of depression among the participants in this study was 26%. Within this group, nearly one-third exhibited mild depression, another third had moderate depression, and the remaining third had severe depression.

In the analysis of the anxiety questionnaire's first stage, the prevalence of anxiety symptoms among the research participants was estimated to be 23.7%. Approximately two-thirds of the patients experienced mild anxiety, while only two patients reported severe anxiety symptoms.

The comparison of average sleep disorder scores at three different times did not reveal any significant differences. However, in the assessment of depression and anxiety, an increasing trend was observed in the second stage, followed by a decreasing trend in the third stage. The average scores for depression and anxiety parameters in the third stage were significantly higher than those in the first stage (Table 2).

Table 3 illustrates the relationship between demographic and clinical factors, anxiety, and depression with sleep quality disorder. The analysis indicated no significant relationship between the patient's age, duration of marriage, woman's occupation, woman's primary education, husband's primary education, duration and type of infertility, history of illness, and use of herbal medicine with sleep disorder. However, the cause of infertility, particularly ovulation disorders and male factors, was associated with higher sleep disorder scores compared to an unknown cause. Additionally, the husband's occupation, specifically farmers and other occupations, was linked to a higher rate of sleep disturbance compared to women whose husbands were employees. In the presence of other variables, anxiety and depression exhibited a positive and significant relationship with sleep quality disorder.

In Table 4, the analysis reveals that there is no significant relationship between the patient's age, duration of marriage, woman's occupation, duration and type of infertility, history of illness, and use of herbal medicine with anxiety and depression. However, women and their spouses with primary education showed higher depression scores compared to those with a university education. Similarly, the cause of infertility, particularly ovulation disorders and male factors, was associated with higher depression scores compared to those with an unknown etiology. Furthermore, the spouse's primary education (compared to university education), the cause of infertility being ovulation disorder (compared to an unknown cause), and the wife's occupation as a farmer and other occupations (compared to being an employee) were linked to a higher level of anxiety.

## 4. Discussion

The objective of this study was to investigate the factors influencing sleep quality in women undergoing IUI.

The prevalence of sleep quality disorder in our study, at 30.5%, aligns closely with Lin et al.'s (2014) findings. Their study on physical symptoms, psychological distress, and sleep disorder in infertile women during IUI revealed that over one-third of women (35%) experienced sleep disorder [11]. Of course, it is worth mentioning that in Shahraki et al.'s study (2017), more than half of infertile women (52%) had poor sleep [14]. Like us, they had used the Pittsburgh sleep disorder and Beck depression questionnaires. Of course, the inclusion criteria for their study were age more than 18 and less than 40 years, candidacy for IVF treatment, and the absence of polycystic ovary, which is different from the inclusion criteria of the present study.

In our study, anxiety prevalence was estimated at 23.7%, with almost two-thirds of the patients experiencing mild anxiety and only 2 patients exhibiting severe anxiety symptoms. Comparatively, a systematic review and meta-analysis by Kiani et al., surveying 5055 infertile women, reported an anxiety prevalence of 36% [21]. Additionally, Omani-Samani et al. investigated infertile women in Tehran, using the GAD-7 questionnaire (different from our study's questionnaire), and declared an anxiety prevalence of 28.3% [22]. The variations in reported prevalence may be influenced by differences in sample sizes, methodologies, and the specific tools employed to assess anxiety.

In our study, the prevalence of depression was 26%, with almost one-third experiencing mild depression, one-third having moderate depression, and one-third having severe depression. A study by Alhassan et al. reported a higher depression prevalence of 62% among infertile women, where Beck's depression test, similar to our study, was employed. Of course, they had investigated 100 infertile women who were not necessarily candidates for a specific type of ART treatment, and perhaps the cultural differences between Ghana and Iran are the reason for the statistical differences in the findings [23]. Another study by Haririan et al. found that 36% of infertile women had mild depression, 10% had moderate depression, 11% had severe depression, and 42% had no depression [24]. Our study's lower prevalence of depression might be influenced by the inclusion of criteria considering the absence of known depression in the last 6 months.

The results indicate that there was no significant difference in the average scores of sleep disorders over the three times. However, there was an interesting trend in depression and anxiety, with an increasing trend in the second stage and then a decreasing trend in the third stage. Importantly, the average scores of depression and anxiety in the third stage were significantly higher than those in the first stage, suggesting that patients entering the treatment cycle may experience an increase in anxiety and depression symptoms. This effect appears to persist for at least 1 month after IUI, as explored in the present study.

These findings emphasize the potential psychological challenges faced by patients undergoing fertility treatments. The inclusion of a psychologist in the infertility treatment team is highlighted as a potential strategy to alleviate psychological distress and enhance the likelihood of a positive outcome in infertility treatment. Unfortunately, similar studies examining the changes in these parameters at different times were not found for direct comparison with the present results.

In the study by Yanik et al., similar to the present study, the PSQI was used, and the number of assessments for women undergoing IVF treatment was comparable to that for women undergoing IUI treatment in the present study, although the timing of their assessments differed. Their results, contrary to those of the present study, showed that women's sleep quality scores gradually decreased as the treatment progressed. In their study, age, low education level, and economic difficulties were associated with poorer sleep quality. Similarly, in the present study, being a farmer's wife was linked to a higher rate of sleep disturbances in women. However, in the present study, no association was observed between age, low education level, or the woman's occupation and sleep disturbances [25].

The results of the study by Li et al. align with the findings of the present study, showing that factors such as anxiety and depression are associated with poor sleep quality. However, Li et al. focused on sleep characteristics before the initiation of ART, whereas the present study examined factors affecting sleep disorders in women undergoing IUI treatment [26].

Similarly, both Lin et al. and Kirca et al. used the PSQI to assess sleep problems in women undergoing oocyte retrieval and IVF. Their findings, in contrast to the present study, indicated that sleep disorders were more prevalent during IVF and embryo transfer. In the present study, there was no significant difference in the mean sleep disorder scores at the three time points assessed [27, 28].

Given the consistency of results among the studies by Yanik, Kirca, and Lin [25, 27, 28] and the difference between their findings and those of the present study, it can be concluded that sleep disorders are more common in women undergoing IVF treatment compared to those undergoing IUI treatment.

The findings of the present study reveal that several demographic factors, including the age of the patient, duration of marriage, woman's occupation, education of the woman and her husband, duration and type of infertility, history of illness, and the use of herbal medicine, did not show a significant relationship with sleep disorder. However, the cause of infertility, specifically ovulation disorder and male factor, was associated with higher sleep disorder scores compared to cases with an unknown cause. Additionally, individuals whose spouses were farmers and had other occupations exhibited a higher rate of sleep disorders compared to women whose spouses were employees.

These results align with a study conducted by Shahraki et al., where no significant relationship between age and sleep disorder was observed [14]. Unfortunately, there is a lack of additional studies in the field to further compare the relationship between demographic factors and sleep disorders.

The findings of our study reveal a significant relationship between the presence of anxiety and depression and a higher score in sleep quality disorder. This aligns with the results of the study conducted by Wang et al. in 2023, where poor sleep quality was identified as an independent risk factor for depression [29]. Similarly, Huang et al. in 2019 found a strong correlation between sleep disorder and depression and anxiety, with depression being a significant predictive factor for sleep disorder [30]. These results are consistent with our study, as all three studies utilized Beck depression and anxiety questionnaires along with Pittsburgh Sleep Disorder Questionnaires.

Additionally, Shahraki et al.'s study in 2017 demonstrated a significant correlation between depression score and sleep disorder score in both infertile and healthy women. This indicates that poor sleep is related to depression in women, whether they are experiencing infertility or are generally healthy [14]. It is worth noting that our study did not evaluate the level of depression in healthy women due to its specific focus on women undergoing IUI.

Our study's findings indicate that there was no significant relationship between various demographic factors such as the patient's age, duration of marriage, woman's occupation, duration and type of infertility, history of illness, and use of herbal medicine with anxiety and depression. However, some specific associations were observed. The primary education of women and their spouses was associated with higher depression scores than those with a university education. Additionally, the cause of infertility, specifically ovulatory disorder and male factors, was linked to higher depression scores compared to cases with an unknown etiology. Similarly, spouses with elementary education, infertility caused by ovulation disorder, and husbands working as farmers or in other occupations were associated with a higher level of anxiety.

It is interesting to note that these findings differ from the results of Wang et al.'s study in 2007, where age over 35 years was identified as an independent risk factor for depression, and a higher education level (primary college degree or higher) was an independent risk factor for anxiety [31]. The differences between these findings could be influenced by various factors such as treatment procedure (IVF/ICSI vs. IUI) or methodological variations between the two studies.

It is reasonable to consider the difference in the questionnaires used, the type of infertility treatment, and cultural variations as potential factors contributing to the statistical differences in findings between studies. The choice of measurement tools and the specific treatments studied can influence the outcomes observed.

The lack of a significant relationship between the duration of infertility and depression in both our study and Shahraki et al.'s study suggests that the duration of infertility may not be a primary factor influencing depression levels [14]. On the other hand, the positive and significant correlation between the level of depression, age of women, and duration of infertility observed in Alhassan et al.'s study may be attributed to cultural and contextual differences. Cultural nuances can significantly impact how individuals experience and express mental health issues. It is noteworthy that, despite these differences, the common observation regarding higher levels of depression among individuals with low or no formal education provides a consistent trend across studies [23].

Considering these variations helps researchers and clinicians contextualize findings and tailor interventions to specific populations, taking into account cultural, social, and educational factors that may influence mental health outcomes.

It is interesting to observe the similarities and differences between your study and Syam's study. The educational background, employment status, and the most common causes and types of infertility in both studies can offer valuable insights into the demographic and clinical characteristics of the populations under investigation. The fact that most women in our study had a university education might indicate a higher level of education among the participants compared to Syam's study, where most women had a high school education. Educational background can influence various aspects of individuals' lives, including their understanding of health-related information, decision-making processes, and access to resources. Additionally, the similarity in the most common causes and types of infertility suggests that these factors might be consistent across different populations undergoing IUI treatment [32].

Understanding these demographic and clinical characteristics helps researchers contextualize their findings and clinicians tailor interventions that are culturally and demographically relevant to the specific population being studied or treated.

### 4.1. Strengths and Limitations

Our study's strength in investigating depression, anxiety, and sleep disorders across three time stages provides valuable insights into the dynamic nature of these psychological and sleep-related factors during the course of IUI treatment. This temporal perspective adds depth to understanding how these aspects evolve and interact over time, contributing to the existing literature on infertility and its psychological implications.

However, it is acknowledged that certain factors with potential confounding effects on sleep quality were not explored in our study, such as caffeine and alcohol consumption, biochemical markers like cortisol, specific drugs used for IUI, semen volume, and the difficulty in performing IUI. These unexplored variables could have influenced the outcomes, and future studies may benefit from considering these factors for a more comprehensive understanding of the relationship between infertility, psychological well-being, and sleep quality.

Also, it is recommended that the authors consider assessing self-efficacy in future research to gain a more comprehensive understanding of the psychosocial factors influencing sleep quality. Given that this study employed repeated-measures ANOVA and GLMs for data analysis, it is advisable that future studies explore the use of generalized estimating equations (GEEs) to better handle temporal or repeated-measures data, thereby enhancing the accuracy of the analysis.

## 5. Conclusion

This study highlighting the prevalence of severe sleep quality disorders among infertile women and the significant impact of depression and anxiety underscores the importance of addressing psychological aspects in infertility treatment clinics. This recommendation aligns with the broader understanding that holistic care, encompassing both physical and mental well-being, is crucial for individuals undergoing fertility treatments. Despite limitations such as a small sample size and possible biases in the data collection process, the present study advances understanding in this field by elucidating the complex interplay between psychological factors and sleep quality within the context of infertility.

## Figures and Tables

**Table 1:** Demographic and clinical characteristics.

Variables	Scale	Total
Age, mean (years)		29.85 (5.75)

Duration of marriage, mean (years)		7.87 (4.22)

Education of women; frequency (%)	Illiterate	0 (0)
	Primary school	13 (9.9)
	Guidance school	17 (13)
	High school	41 (31.3)
	University	60 (45.8)

Education of their husbands; frequency (%)	Illiterate	1 (0.8)
	Primary school	7 (5.4)
	Guidance school	28 (21.5)
	High school	43 (33.1)
	University	51 (39.2)

Occupation of women; frequency (%)	Employee	22 (16.9)
	Unemployed	108 (83.1)

Occupation of their husbands; frequency (%)	Unemployed	0 (0)
	Manual worker	27 (20.6)
	Employee	31 (23.7)
	Freelance job	58 (44.3)
	Farmer	7 (5.3)
	Other jobs	8 (6.1)

Duration of infertility; mean (years)		4.85 (3.39)

Cause of infertility; frequency (%)	Ovulation disorders	44 (37.3)
	Tubal	3 (2.5)
	Uterine	0 (0)
	Male factor	21 (17.8)
	Both	21 (17.8)
	Unknown	29 (24.6)

Type of infertility; frequency (%)	Primary	78 (72.9)
	Secondary	29 (27.1)

History of diseases; frequency (%)	No history of illness	109 (87.9)
	Rheumatoid arthritis	3 (2.4)
	Cervical ulcer	1 (0.8)
	Abortion	3 (2.4)
	Hypothyroidism	2 (1.6)
	Infection	3 (2.4)
	Fibroma	1 (0.8)
	One-way blockage of tubal	2 (1.6)

Taking herbal medicine; frequency (%)	No	103 (85.1)
	Yes	18 (14.9)

**Table 2:** Average sleep disorder, depression, and anxiety in three stages.

		**Positive mean (SD)**	**Negative mean (SD)**	**Between-effect** **p** **value**

Sleep disorder^a^	Stage 1	7.06 (4.11)	7.84 (5.73)	0.577
	Stage 2	8.17 (4.49)	9.68 (6.77)	0.362
	Stage 3	9.28 (4.59)	8.75 (6.81)	0.753

Within-effect *p* value		**0.042**	**0.001**	

Depression^b^	Stage 1	11.17 (8.54)	10.13 (10.58)	0.516
	Stage 2	8.39 (7.31)	13.01 (12.26)	0.375
	Stage 3	9.33 (7.93)	12.26 (12.62)	0.458

Within-effect *p* value		0.108	**0.001**	

Anxiety^c^	Stage 1	5.50 (5.08)	6.72 (7.66)	0.694
	Stage 2	7.11 (7.16)	9.05 (8.80)	0.123
	Stage 3	6.78 (5.96)	8.38 (8.80)	0.343

Within-effect *p* value		0.491	**0.002**	

*Note:p*-value for Box's test of equality of covariance matrices and Mauchly's test of sphericity assumptions. The within-group effects based on repeated measures ANOVA indicate that sleep disorder (Pos. group: *p* = 0.042), sleep disorder (Neg. group: *p* = 0.001), depression (Neg. group: *p* = 0.001), and anxiety (Neg. group: *p* = 0.002) are statistically significant.

^a^ *p* = 0.553, *p* = 0.176.

^b^ *p* = 0.058, *p* = 0.230.

^c^ *p* = 0.262, *p* = 0.076.

**Table 3:** The relationship between demographic and clinical factors, anxiety, and depression with sleep disorder (based on the first-stage questionnaire).

Variables	Scale	Sleep disorder	
		*β* (SE)	*p*
Age	—	0.075 (0.06)	0.211

Duration of marriage	—	0.013 (0.07)	0.861

Education of women (reference: university)	Primary school	1.139 (1.26)	0.369
	Guidance school	−0.069 (1.12)	0.951
	High school	−0.050 (0.74)	0.497

Education of women (reference: university)	Primary school	−0.161 (1.75)	0.927
	Guidance school	−0.328 (0.87)	0.707
	High school	−0.388 (0.79)	0.624

Occupation of women (reference: unemployed)	—	−0.601 (0.94)	0.525

Occupation of their husbands (reference: employee)	Manual worker	−0.853 (0.95)	0.372
	Freelance job	0.466 (0.77)	0.549
	Farmer	**2.78 (1.36)**	**0.041**
	Other jobs	**3.03 (1.49)**	**0.042**

Duration of infertility	—	0.101 (0.093)	0.278

Cause of infertility (reference: unknown)	Ovulation disorders	**1.851 (0.80)**	**0.022**
	Fallopian tube	1.944 (2082)	0.491
	Male factor	**1.05 (2.94)**	**0.005**
	Both	1.73 (0.97)	0.077

Type of infertility (reference: secondary)	—	−0.067 (0.72)	0.926

History of diseases (reference: no history of illness)	—	0.441 (1.08)	0.684

Taking herbal medicine (reference: no consumption)	—	−1.239 (0.93)	0.186

Anxiety	—	**0.186 (0.03)**	**< 0.001**

Depression	—	**0.158 (0.03)**	**< 0.001**

*Note:* Occupation of women (farmer and other jobs vs. employee: *p* = 0.041 and *p* = 0.042), cause of infertility (ovulation disorders and male factor vs. unknown: *p* = 0.022 and *p* = 0.005), anxiety (*p* < 0.001), and depression (*p* < 0.001).

**Table 4:** Examining the relationship between demographic and clinical factors with anxiety and depression (based on the first-stage questionnaire).

Variables	Scale	Anxiety		Depression	
		*β* (SE)	*p*	*β* (SE)	*p*
Age	—	−0.091 (0.13)	0.506	−0.282 (0.17)	0.111

Duration of marriage	—	0.055 (0.17)	0.758	0.052 (0.23)	0.822

Education of women (reference: university)	Primary school	5.496 (3.07)	0.074	**7.868 (3.97)**	**0.048**
	Guidance school	1.925 (2.65)	0.468	5.625 (3.42)	0.101
	High school	−0.144 (1.82)	0.937	2.58 (2.36)	0.274

Education of their husbands (reference: university)	Primary school	**9.455 (4.47)**	**0.035**	**13.90 (5.72)**	**0.015**
	Guidance school	0.216 (2.07)	0.917	4.671 (2.64)	0.078
	High school	−0.474 (1.90)	0.804	0.492 (2.43)	0.840

Occupation of women (reference: unemployed)	Employee	−2.572 (2.46)	0.296	−5.198 (3.18)	0.102

Occupation of their husbands (reference: employee)	Manual worker	2.850 (2.26)	0.207	4.743 (3.13)	0.131
	Freelance job	1.715 (1.89)	0.365	2.023 (2.62)	0.442
	Farmer	**9.909 (3.22)**	**0.002**	8.439 (4.47)	0.059
	Other jobs	**10.90 (3.46)**	**0.002**	5.873 (4.81)	0.223

Duration of infertility	—	0.404 (0.23)	0.076	0.501 (0.29)	0.092

Cause of infertility (reference: unknown)	Ovulation	**5.458 (2.02)**	**0.007**	**6.282 (2.64)**	**0.017**
	Tubal	−3.650 (7.46)	0.625	−3.150 (9.74)	0.747
	Male factor	3.350 (2.61)	0.208	**8.433 (3.47)**	**0.015**
	Both	1.475 (2.44)	0.546	2.413 (3.19)	0.450

Type of infertility (reference: secondary)	—	1.604 (1.81)	0.376	0.905 (2.37)	0.703

History of diseases (reference: no history of illness)	—	−2.268 (2.69)	0.399	1.616 (3.52)	0.646

Taking herbal medicine (reference: no consumption)	—	−3.135 (2.36)	0.184	−2.838 (3.10)	0.360

*Note:* Anxiety: Education of their husbands (primary school vs. university: *p* = 0.035), occupation of their husbands (farmer and other jobs vs. employee: *p* = 0.002 and *p* = 0.002), and cause of infertility (ovulation disorders vs. unknown: *p* = 0.007). Depression: Education of women (primary school vs. university: *p* = 0.048), education of their husbands (primary school vs. university: *p* = 0.015), and cause of infertility (ovulation disorders and male factor vs. unknown: *p* = 0.017 and *p* = 0.015).

## Data Availability

The dataset analyzed during the current study is available from the corresponding author upon reasonable request.

## Supporting Information

In the separate analysis of the seven sleep quality domains, the majority of patients showed no impairment in sleep duration, sleep efficiency, and daytime functioning and were also not using sleep medications. In the domains of subjective sleep quality and sleep disturbances, most patients exhibited mild impairment. The majority of patients experienced mild to moderate impairment in sleep latency. Only one patient showed severe impairment in both sleep medication use and sleep disturbances.
