# Supplementary material for: A Cohort Study on Factors Affecting Sleep Quality in Women Undergoing Intrauterine Sperm Insemination (IUI) Treatment
Source: Obstet Gynecol Int. 2025 Jul 28;2025:4749954. doi: 10.1155/ogi/4749954 (PMC12321408; doi:10.1155/ogi/4749954)
Supplement: Supporting Information — Additional supporting information can be found online in the Supporting Information section. [file 4749954.f1.docx]

In the separate analysis of the seven sleep quality domains, the majority of patients showed no impairment in sleep duration, sleep efficiency, and daytime functioning, and were also not using sleep medications. In the domains of subjective sleep quality and sleep disturbances, most patients exhibited mild impairment. The majority of patients experienced mild to moderate impairment in sleep latency. Only one patient showed severe impairment in both sleep medication use and sleep disturbances.

Seven Sleep Quality Domains *(Based on Phase 1 Questionnaire Data)*

| **Sleep Disorder Domains** | scale | frequency | percentage |
| --- | --- | --- | --- |
| **Sleep Disorder Domains** | No disorder | 91 | **69/5** |
|  | With disorder | 40 | 30/5 |
| **Subjective Sleep Quality** | No disorder | 42 | 32/1 |
|  | Mild disorder | 82 | **62/6** |
|  | Moderate disorder | 5 | 8/3 |
|  | Sever disorder | 2 | 1/5 |
| **Sleep Latency (Time to Fall Asleep)** | No disorder | 22 | 17/5 |
|  | Mild disorder | 45 | 35/7 |
|  | Moderate disorder | 48 | **38/1** |
|  | Sever disorder | 11 | 8/7 |
| **Sleep Duration** | No disorder | 72 | **60/5** |
|  | Mild disorder | 27 | 22/7 |
|  | Moderate disorder | 14 | 11/8 |
|  | Sever disorder | 6 | 5 |
| **Sleep Efficiency** | No disorder | 92 | **81/4** |
|  | Mild disorder | 14 | 12/4 |
|  | Moderate disorder | 4 | 3/5 |
|  | Sever disorder | 3 | 2/7 |
| **Sleep Disturbances** | No disorder | 17 | 13 |
|  | Mild disorder | 98 | **74/8** |
|  | Moderate disorder | 15 | 11/5 |
|  | Sever disorder | 1 | 0/8 |
| **Use of Sleep Medication** | No disorder | 124 | **94/7** |
|  | Mild disorder | 4 | 3/1 |
|  | Moderate disorder | 2 | 1/5 |
|  | Sever disorder | 1 | 0/8 |
| **Daytime Dysfunction** | No disorder | 72 | **55/8** |
|  | Mild disorder | 48 | 37/2 |
|  | Moderate disorder | 9 | 7 |
